# Dualistic MADS-box evolution forged legume diversity post-WGD

**DOI:** 10.3389/fpls.2025.1740598

**Published:** 2026-01-15

**Authors:** Haiyang Nan, Xinxin Chen, Jiajia Zhang, Mingyang Zou, Erxuan Shang, Xinyi Guo, Kun Kou

**Affiliations:** 1 Engineering Research Center of Agricultural Microbiology Technology, Ministry of Education & Heilongjiang Provincial Key Laboratory of Plant Genetic Engineering and Biological Fermentation Engineering for Cold Region & Key Laboratory of Molecular Biology, College of Heilongjiang Province & School of Life Sciences, Heilongjiang University, Harbin, China; 2 College of Information Engineering, East University of Heilongjiang, Harbin, China

**Keywords:** asymmetric expression, legume, MADS-box, SSD, WGD

## Abstract

The MADS-box gene family plays a central role in plant development and adaptation, yet its evolutionary history in legumes is remarkably complex. In this study, we performed a pangenomic analysis across 52 legume species, identifying 4,872 MADS-box genes and reconstructing their phylogeny into 16 subfamilies. Our analysis uncovered a pervasive dualistic evolutionary model driven by distinct duplication mechanisms. Structurally, the genes fall into two categories: the compact, intron-poor Type I and the complex, intron-rich Type II. We demonstrate that whole-genome duplication (WGD) serves as the major driver (42.2%) behind the expansion of the conserved core genome, which includes key floral regulators such as the “ABCDE model” genes. These WGD-derived genes are under strong purifying selection, thereby ensuring developmental stability. In contrast, small-scale duplication (SSD) fuels the expansion of the dynamic periphery, primarily composed of Type I genes and stress-responsive clades, which evolve under relaxed selection and promote lineage-specific innovation—as strikingly exemplified by the massive tandem expansion of the SVP subfamily in Prosopis. Pangenome analysis confirmed that WGD-derived genes were enriched in the conserved core genome, underpinning essential functions, whereas SSD-derived genes dominated the variable genome and acted as a source of genetic novelty. Transcriptome analysis in soybean identified four organ-specific expression modules, predominantly comprising Type II core genes. Under biotic and abiotic stress, WGD-derived gene pairs exhibited prominent asymmetric expression. The expression divergence was validated by qRT-PCR. Overall, our findings establish a unified framework for MADS-box gene evolution in legumes, illustrating how divergent duplication mechanisms and selective pressures have collectively shaped a gene family critical to both evolutionary innovation and developmental stability.

## 1 Introduction

Whole-genome duplication (WGD), a form of macro-mutation, is a primary engine of evolution in eukaryotes, and it is so pervasive in plants that all extant angiosperms are considered to be paleopolyploids (Clark and Donoghue, 2018; Soltis et al., 2015). This explosive, genome-wide event stands in stark contrast to the continuous, localized “micro-mutations” of small-scale duplications (SSDs)—a collection of processes including tandem, proximal, dispersed, and transposed duplications—which typically add genes one at a time (Freeling, 2009; Mascagni, et al., 2021). While SSDs provide a steady, manageable stream of raw material, WGD is not a simple doubling of gene content but rather the start of a dynamic WGD-fractionation cycle, in which most duplicated genes are eventually lost over millions of years (Hong et al., 2021; Soltis et al., 2015). Indeed, without extensive fractionation, some plant genomes would be ten times their current size (Comai, 2005). The surviving gene duplicates, however, provide vast raw material for innovation through neofunctionalization or subfunctionalization (Guo et al., 2013; Konrad et al., 2011; Xue and Fu, 2009). Yet, WGD imposes a severe genomic shock, often leading to reduced fertility and instability, making most polyploidization events short-lived evolutionary dead-ends (Shimizu, 2022). For those that persist, a fundamental challenge arises: how to manage massive gene redundancy while maintaining the integrity of complex regulatory networks. The “gene dosage balance hypothesis” posits that genes encoding components of stoichiometric complexes, such as transcription factor networks, are particularly sensitive to such disruption, creating a strong selective pressure to preserve their relative quantities (Birchler and Veitia, 2010, 2014; Conant et al., 2014; Liang and Fernandez, 2008; Shi et al., 2020). This establishes a fundamental tension for any post-polyploid lineage: how to resolve the conflict between the immense creative potential of gene duplication and the stringent homeostatic constraints required for survival?

The Fabaceae (legume) family represents a spectacular evolutionary success, ranking as the third-largest angiosperm family with nearly 20,000 species and dominating ecosystems worldwide (Yu et al., 2025; Zhao et al., 2021a). Its rapid diversification is intimately linked to a complex history of polyploidy, highlighted by a major WGD event shared among the Papilionoideae subfamily occurring approximately 59 million years ago (Mya), shortly after the Cretaceous-Paleogene (K-Pg) mass extinction (~66 Mya) (Koenen et al., 2021; Vanneste et al., 2014). This timing suggests a compelling hypothesis: the genomic plasticity conferred by WGD provided a critical adaptive advantage, enabling ancestral legumes to colonize the numerous ecological niches left vacant by the extinction event. This scenario raises a fundamental question: What were the specific genomic features that allowed the ancestral legume to harness the genetic instability of WGD, leading not to extinction but to an explosion of diversity and key innovations?

A key to this success lies in the evolutionary dynamics of master regulatory gene families, chief among them the MADS-box family. Named after its founding members from fungi (*MCM1*), plants (*AGAMOUS*, *DEFICIENS*), and animals (*SRF*), this ancient transcription factor lineage orchestrates nearly all major developmental transitions in plants (Goyal et al., 2023). Their function relies on the assembly of multiprotein complexes that bind to specific CArG-box DNA motifs (CC(A/T)6GG) in target genes (Airoldi and Davies, 2012; Melzer and Theissen, 2009; Shen et al., 2021). This mechanism is famously exemplified by the “floral quartet model”, which provides the molecular basis for the “ABCDE model” of flower development (Ali et al., 2019; Liu et al., 2010; Melzer and Theissen, 2009). Critically, the plant MADS-box family is partitioned into two ancient lineages with distinct structures (Bartlett, 2017; Ng and Yanofsky, 2001). The Type II (MIKC) genes possess a characteristic four-domain structure: a MADS (M) domain for DNA binding, an Intervening (I) domain for dimerization specificity, a C-terminal (C) domain for transcriptional activation, and a Keratin-like (K) domain (Hu et al., 2023; Thangavel and Nayar, 2018; Zhao et al., 2021a). The evolution of this K domain, which acts as a scaffold for forming higher-order complexes, was a milestone that enabled the combinatorial control necessary for complex morphologies (Ambrose et al., 2021; Manzoor et al., 2024). In contrast, the structurally simpler and less-studied Type I genes generally lack this K domain and play critical but enigmatic roles in reproduction, particularly in gametophyte and endosperm development (Colombo et al., 2008; De Bodt et al., 2003; Nam et al., 2004).

The ancient structural dichotomy between Type I and Type II MADS-box genes provided a critical genomic template for the legume-specific WGD. We hypothesized that this WGD event acted as a powerful selective filter on these pre-existing lineages. Specifically, we posited that WGD preferentially preserved the complex, dosage-sensitive Type II core to maintain stability, while SSD fueled the expansion of the streamlined Type I periphery to drive innovation. In this study, we undertook a large-scale phylogenomic investigation of 4,872 MADS-box genes across 52 Fabaceae species to reconstruct their duplication histories and trace their evolutionary trajectories. By integrating this deep evolutionary framework with functional insights from expression analyses in *Glycine max*, this study aims to dissect the contrasting roles of WGD versus SSD in shaping this pivotal gene family.

## 2 Materials and methods

### 2.1 Genome data acquisition and species tree reconstruction

We retrieved the genome assemblies and protein annotations for 52 Fabaceae species from multiple public databases, including NCBI, plantGIR (Liu et al., 2024) and LGRPv2 (Yu et al., 2025). Detailed information was provided in **Supplementary Table S1**. To establish a robust species evolutionary framework, a consensus topology (branching structure) was obtained from the TimeTree 5 web server (Kumar et al., 2022) and LGRPv2 (Yu et al., 2025). Documented WGD and whole genome triplications (WGT) events were manually annotated onto the species tree based on published literature (Jiao et al., 2012; Yu et al., 2025; Zhang et al., 2020; Zhao et al., 2021b).

### 2.2 Identification of MADS-box gene family in Fabaceae

The HMM profile of the conserved SRF-TF domain (PF00319) was used to search against the proteomes of all 52 Fabaceae species. To provide a broader evolutionary context, we also searched the proteomes of selected outgroup species: *Polygala tenuifolia*, *Vitis vinifera*, *Amborella trichopoda*, *Nymphaea colorata*, *Arabidopsis thaliana*, and *Oryza sativa* (**Supplementary Table S1**). All searches were conducted using HMMER (v3.4) with a cutoff E-value cutoff of 1×10^-5^ (Potter et al., 2018). To ensure high accuracy, all candidate sequences were manually curated. Sequences shorter than 100 amino acids were removed, and the presence of the MADS domain in the remaining candidates was verified using the NCBI Conserved Domain Database (NCBI-CDD) web server.

### 2.3 Phylogenetic analysis and subfamily classification

To elucidate the evolutionary relationships within the MADS-box family, we performed a multi-step phylogenetic analysis. The full-length amino acid sequences of all identified MADS-box proteins were aligned using MAFFT (v7.505) (Katoh and Standley, 2013) and then trimmed to remove poorly aligned regions using trimAl (v1.4) (Capella-Gutiérrez et al., 2009). A maximum likelihood (ML) phylogenetic tree was constructed from the trimmed alignment using FastTree (v2.1.11) with the JTT (Jones-Taylor-Thornton) protein substitution model. Local branch support was assessed using the Shimodaira-Hasegawa-like (SH-like) method, based on 1,000 resamples, as implemented in FastTree (Price et al., 2009). Based on the topology of the phylogenetic tree and established domain architectures from foundational studies (Kaufmann et al., 2005; Parenicová et al., 2003). Tree visualization was done with Evolview (Subramanian et al., 2019).

### 2.4 Gene structure and motif analysis

Gene structure statistics, including gene length, amino acid length, intron length, intron/exon number were retrieved from genomic annotation GFF3 files for each species. To investigate the conservation and divergence of protein architecture, we analyzed the conserved motifs within each MADS-box subfamily using the MEME suite (v5.5.8) (Bailey et al., 2015). The analysis was set to identify a maximum of 5 motifs and only those that appear in over 50% of all sequences were selected for visualization.

### 2.5 Analysis of duplication events and selection pressure

The protein sequences were aligned using Diamond v2.0.5.143 in blastp mode with a E-value cutoff 0.001 and max-target-seqs 5 (Buchfink et al., 2021). Synteny analysis was conducted for each species using MCScanX with the following parameters: a minimum of colinear genes were required to define a block (-s 5), and the maximum gene gap allowed between collinear genes was set to (-m 25) (Wang et al., 2012). Based on the synteny information and genomic locations, duplicated gene pairs were classified into five types: WGD, tandem, proximal, dispersed, and transposed, using the DupGen-finder (v1.0.0) (Qiao et al., 2019). The rates of synonymous (*Ks*) and non-synonymous (*Ka*) substitutions were calculated using KaKs_Calculator (v2.0). To mitigate the impact of noise and artifacts on evolutionary rate estimates, only gene pairs with *Ks* values < 2 and *P*-Value < 0.05 were included in subsequent analyses. The ratio Ka/Ks was then used to infer the selection pressure acting on these duplicated genes.

### 2.6 Pangenome analysis of MADS-box subfamilies

To assess the conservation and dispensability of MADS-box subfamilies across the Fabaceae, we conducted a pangenome analysis. For each subfamily, protein sequences from all 52 species were clustered into orthologous groups (OGs) using CD-HIT (v4.8.1) a 40% sequence identity threshold (Fu et al., 2012). The resulting clusters were categorized into four frequency-based groups according to established pangenome nomenclature (Tong et al., 2025): (i) core (present in all 52 Fabaceae species), (ii) soft-core (present in >90% of species), (iii) shell (present in 10%–90% of species), (iv) cloud (present in <10% of species).

### 2.7 Gene expression analysis in soybean

To investigate the functional divergence of MADS-box genes, particularly among duplicated pairs, we analyzed a large-scale RNA-seq dataset for soybean (*Glycine max*). We retrieved the expression profiles of 3,638 RNA-seq samples of soybean derived from various tissues and abiotic and biotic stresses from the soybean RNA-seq Database (https://plantrnadb.com/soybean/) (Yu et al., 2022) (**Supplementary Table S2**). FPKM values were extracted for all identified soybean MADS-box genes. These values were used to analyze tissue-specific expression patterns and to compare the expression divergence between duplicated gene pairs derived from WGD and SSD events.

To identify organ-specific expression modules, hierarchical clustering was performed using the pheatmap package in R. Prior to clustering, FPKM values were log2-transformed and standardized using *z*-scores to highlight expression trends rather than absolute abundance. A distance matrix was computed using the Euclidean method, and clustering was conducted using the Ward’s minimum variance method (Ward.D2). The resulting dendrogram was cut to define four distinct expression modules.

### 2.8 Plant cultivation, treatments, RNA isolation, and qRT-PCR

To validate the expression patterns, soybean seeds were germinated on moist paper for 5 days and then transferred into a light chamber under a 16h:8h, light: dark photoperiod with 60% relative humidity at 25 °C. The seedlings were grown in pots containing a 2:1 mixture of forest soil: vermiculite for 2 weeks. Select plants with uniform growth and divide them into four groups. For drought treatment, the seedlings roots were rinsed with water to remove adherent soil without damaging root hairs. Then excess surface water was blotted using filter papers for the induction of rapid drought for 0 (CK), 1, 3, 8 and 12 hours. For NaCl treatment, the seedlings were treated separately with 1/2 Hoagland nutrient solution, 150 mM NaCl solution and 200 mM NaCl solution on different periods (0, 1, 3, 8 and 12 hours), respectively. Leaf samples are collected into test tubes, rapidly frozen with liquid nitrogen, and subsequently used for RNA extraction. Total RNA was extracted using the Ultrapure RNA Kit (CWBIO, China), which includes TRIzon Reagent as a key component, and cDNA was synthetized with M-MLV reverse transcriptase kit (Takara). Quantitative real-time polymerase chain reaction (qRT-PCR) was performed with SYBR Premix Ex (TaKaRa) on the Roche Light Cycler 480 system (Roche, Germany) with PCR kit (Roche, Germany). The soybean endogenous gene *TUBULIN* (*Glyma.05G157300*) was used as an internal control, and the relative expression levels of examined genes were calculated using the 2^-ΔΔCt^ method (Livak and Schmittgen, 2001). Three biological replications were performed in each test. The primers are listed in **Supplementary Table S3**.

## 3 Results

### 3.1 Identification and phylogenetic classification of MADS-box in Fabaceae

**Figure 1:**
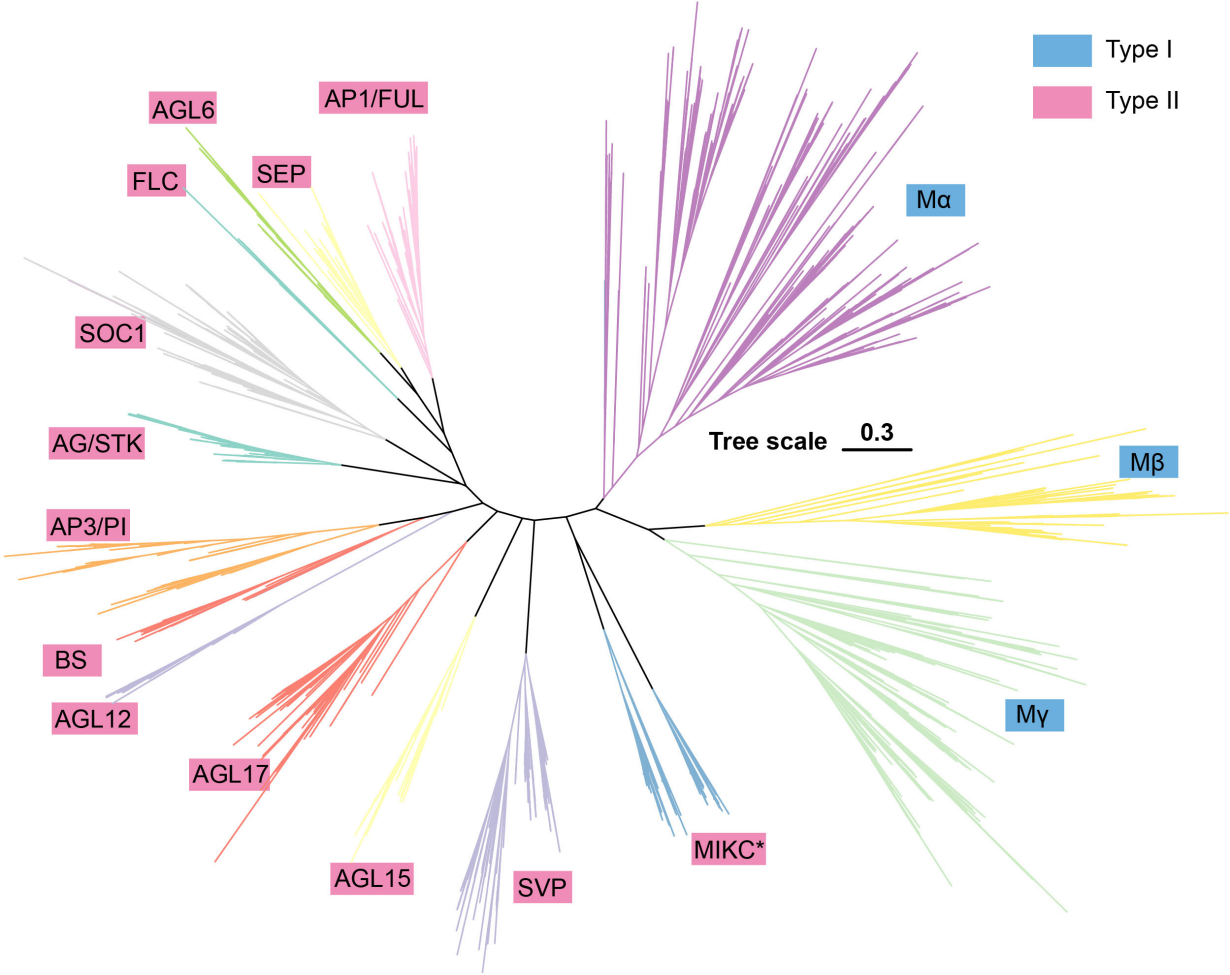
Phylogenetic dichotomy partitions the Fabaceae MADS-box family into type I and type II lineages.

To systematically map the MADS-box gene family across the Fabaceae, we conducted genome-wide search in 52 species of legumes that represent three major subfamilies: 38 species from the Papilionoideae subfamily, 11 from the Caesalpinioideae, and 3 from the Cercidoideae (**Supplementary Table S4**). A total of 4,872 high-confidence MADS-box genes were identified, comprising 2,032 Type I and 2,840 Type II (MIKC) members (**Supplementary Table S4**). To elucidate the evolutionary relationships more clearly, we included several evolutionarily significant outgroup species in our analysis, encompassing, including *Polygala tenuifolia*, *Vitis vinifera*, *Amborella trichopoda*, *Nymphaea colorata*, *Arabidopsis thaliana*, *Oryza sativa*, and conducted identification for these species as well (**Supplementary Tables S1**, **S4**). The resulting phylogeny clearly partitions the family into 16 subfamilies (**Figure 1**). The Type I lineage is the largest branch, comprising the Mα, Mβ, and Mγ clades, with Mβ and Mγ showing a closer relationship. The Type II lineage consists of 13 distinct clades, with clear clustering observed among core floral development genes. Among them, AG/STK, SOC1, FLC, AGL6, SEP, and AP1/FUL are clustered together, suggesting a closer phylogenetic relationship. Additionally, the AP3/PI, BS, and AGL12 are also clustered together, indicating a closer relationship. The other clades in Type II are relatively independent, indicating that they may have undergone independent evolution.

### 3.2 Deep structural dichotomy defines the MADS-box family

Beyond their phylogenetic placement, a profound structural dichotomy between Type I and Type II genes became immediately apparent, providing a physical basis for their divergent evolutionary fates.

**Figure 2:**
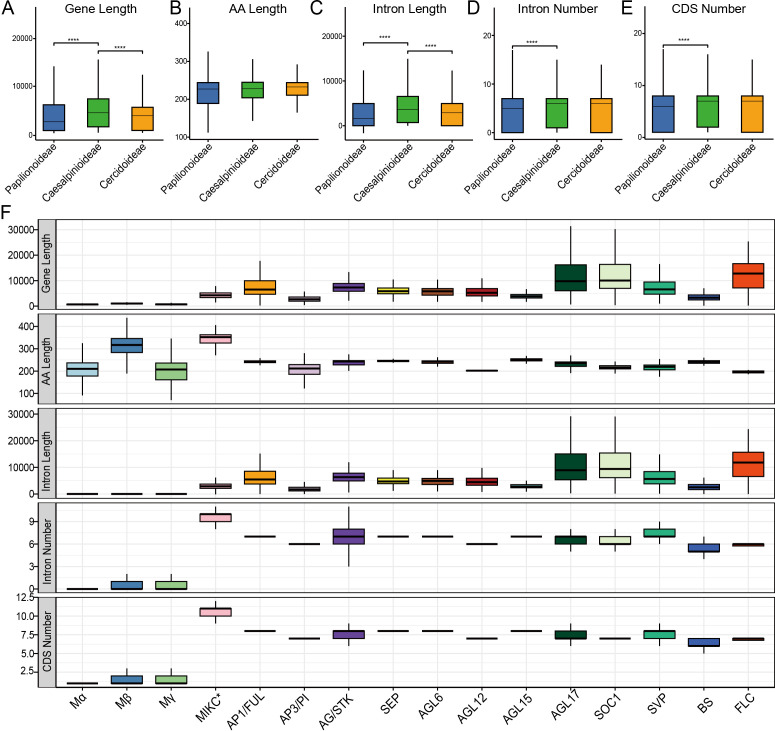
Structural dichotomy distinguishes type I and type II MADS-box genes. Boxplot gene length, Amino acid (AA) length, intron length, intron number and CDS number among three species clades **(A–E)** and subfamilies **(F)**. Statistical significance was assessed using a t.test. Asterisks indicate statistical significance (*P* < 0.001). Non-significant comparisons are not marked.

We observed significant variation in gene architecture across both lineages and subfamilies (**Figure 2**). A comprehensive structural survey of the MADS-box gene family among 52 legume species uncovered evolution patterns among different MADS-box subfamilies in three subfamilies (Papilionoideae, Caesalpinioideae and Cercidoideae) (**Figures 2A–E**). Gene length exhibits significant variation across different lineages, with Caesalpinioideae possessing longer gene (**Figure 2A**) and intron lengths (**Figure 2C**). This suggests that differences in intron length among lineages may contribute to variations in gene length. In contrast, amino acid sequence length shows no significant differences among the three lineages (**Figure 2B**), highlighting the high conservation of coding regions within the Fabaceae. Regarding intron number (**Figure 2D**) and CDS number (**Figure 2E**), Cercidoideae consistently have slightly higher values than Papilionoideae, underscoring the divergent gene structures present in different species lineages.

Type II genes are structurally complex, with an average length of 8,325 bp and typically containing 6 to 8 introns; some subfamilies, such as AGL17 (12,673 bp), SOC1 (13,148 bp), and FLC (12,916 bp), are particularly long due to extensive introns (**Figure 2F**). In stark contrast, Type I genes are structurally streamlined, with averaging over 9 introns per gene. Their average gene length is only 1,251 bp, and a remarkable 68.6% of genes are intronless and 91.04% have one or fewer introns. This pattern holds across Type I subfamilies, with 75.38% of Mα genes, 65.58% of Mβ genes, and 61.20% of Mγ genes lacking introns entirely (**Figure 2F**).

**Figure 3:**
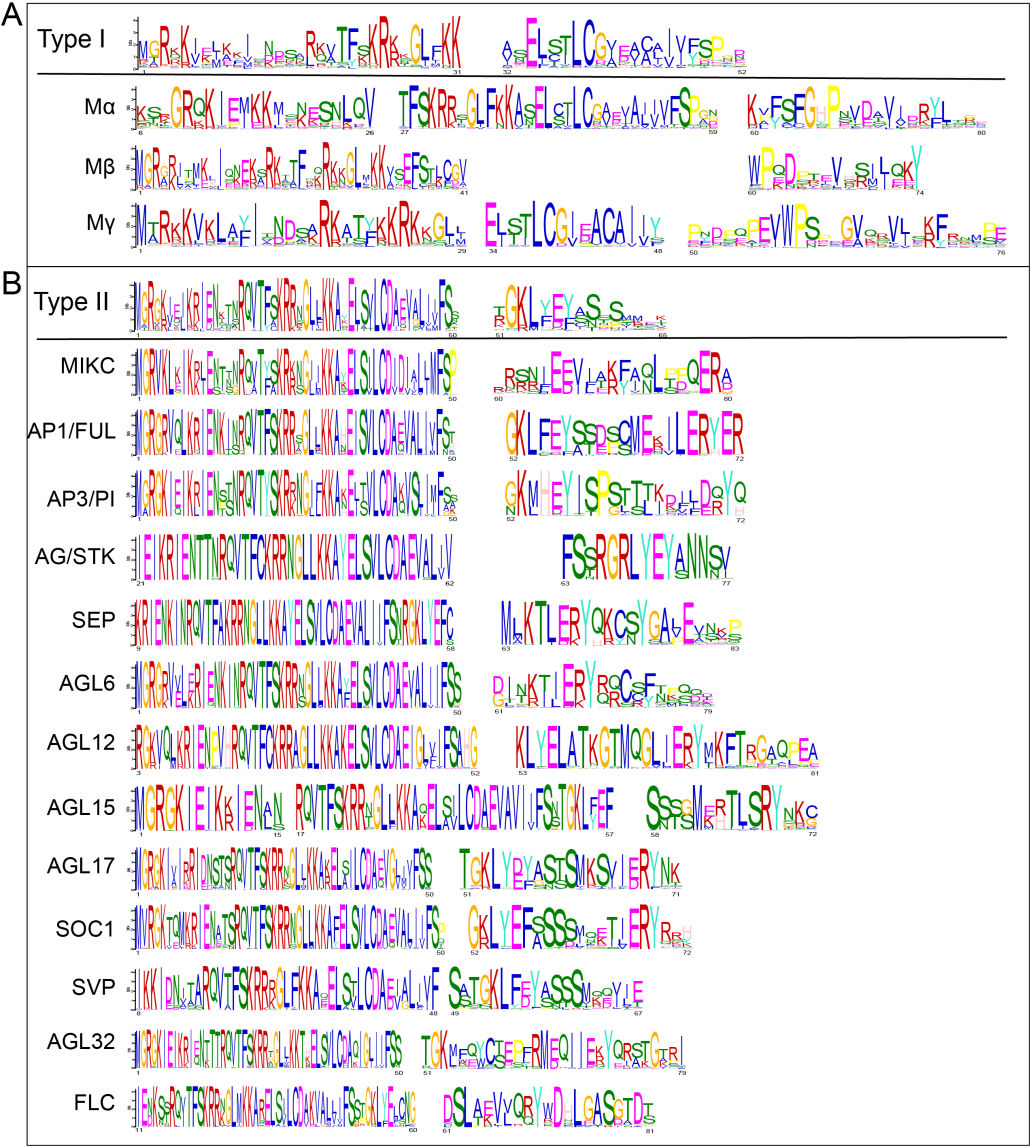
Conserved motif analysis reinforces the divergence of type I and type II MADS domains. Conserved MADS-domain motifs for **(A)** Type I subfamilies, and **(B)** Type II subfamilies. Sequence logos illustrate amino acid conservation; schematics below show motif positions. The statistical significance (E-value) for each motif is indicated next to its label in the figure key.

Motif analysis of the MADS domain further reinforces this dichotomy (**Figure 3**). Key MADS domains SRF-like domain (CDD:238166) were identified in Type-I (**Figure 3A**) and MEF2-like domain (CDD:238165) were identified in Type-II (**Figure 3B**). The MADS domain, typically ranging from 71 to 83 amino acids in length, is highly conserved and merits a detailed analysis of its sequence. All Type I proteins contain a highly conserved SRF-like domain (CDD:238166) characterized by two consensus motifs: “MGRKKIELKKISNDSARKVTFSKRKKGLFKK” and “ASELSTLCGVEACAIVFSPGD”. All Type II proteins feature a MEF2-like domain (CDD:238165), also defined by two motifs, but their primary consensus sequences “MGRGKIEJKRIENKTNRQVTFSKRRNGLLKKAYELSVLCDAEVALIIFSS” and “TGKLYEYASSSMMEK” are more highly conserved than its Type I counterpart and shows distinct positional variations among subfamilies. For example, in the AG/STK subfamily, it starts at the 21st amino acid from the N-terminus, while in the FLC subfamily, it begins at the 11th amino acid. In the AGL6 subfamily, there are 10 amino acids with higher divergence between the two motifs. It suggests fine-scale structural evolution even within this conserved domain.

### 3.3 Dynamic history of lineage-specific expansion and gene loss

**Figure 4:**
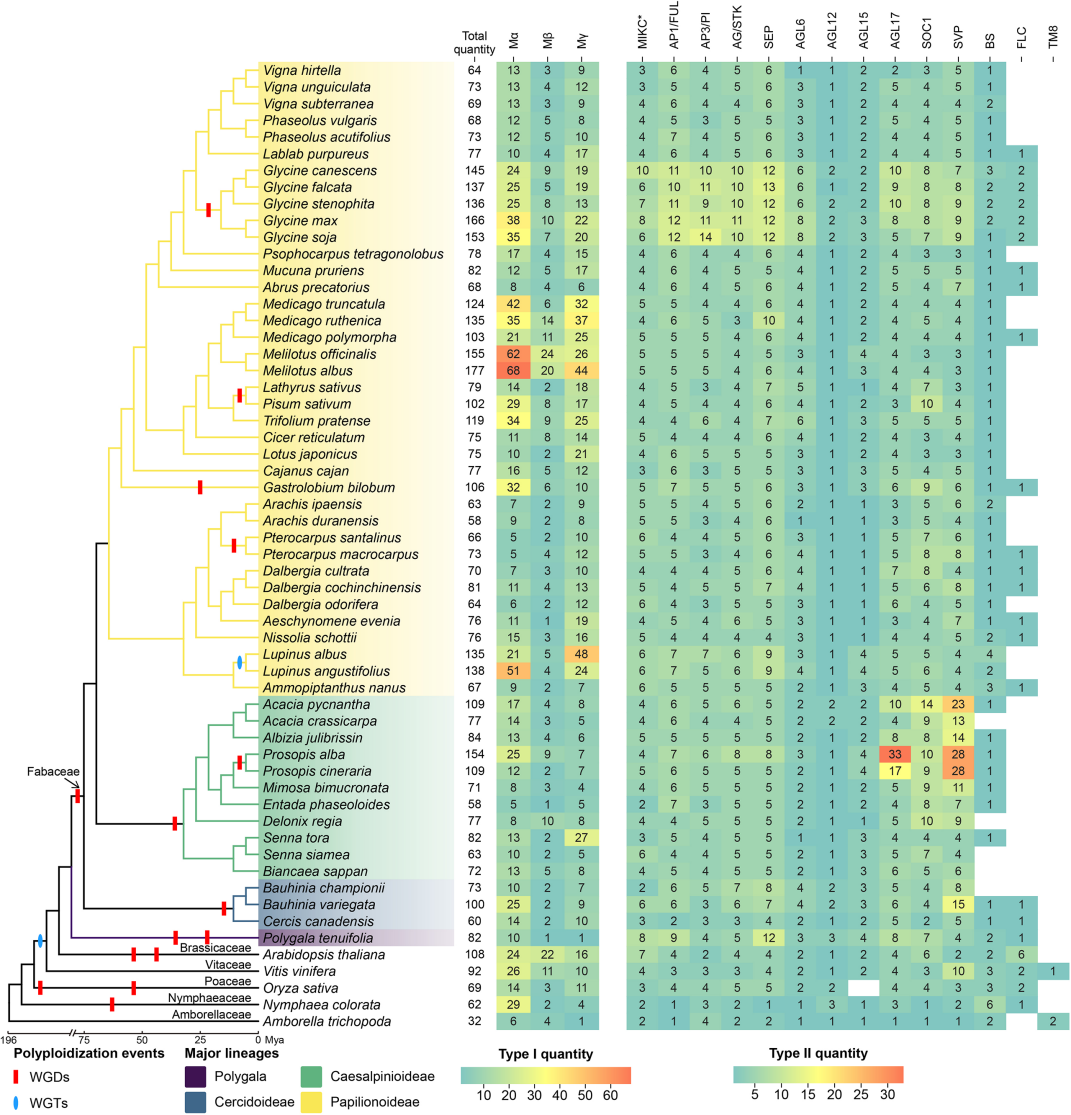
The MADS-box family exhibits a dynamic history of expansion and contraction across the Fabaceae. MADS-box gene family size mapped onto a species cladogram for 52 Fabaceae species. Heatmaps show total gene count and counts per subfamily, revealing wide variation, lineage-specific expansions, and frequent gene loss. The outgroup species Polygala tenuifolia (Polygalaceae) is highlighted in a distinct color to visually root the Fabaceae clade.

The total MADS-box gene count varies dramatically across the Fabaceae, from 60 in *Cercis canadensis* to 177 in *Melilotus albus*, reflecting a dynamic evolutionary history of gene gain and loss (**Figure 4**). Gene number expansion is often linked to polyploidy; the recent WGD in *Glycine* genus, which contrasts with other species. For example, the Type-I subfamily in soybean comprises 70 MADS-box genes, with the Mα, Mβ, and Mγ subfamilies containing 38, 10, and 22 genes, respectively. The Type-II subfamily has a total of 96 genes, with the number of genes in different subfamilies ranging from 2 to 12. Furthermore, we observed specific expansions in some subfamilies in some species. Specifically, the number of genes in the Type-I Mα and Mγ subfamilies in *Glycine*, *Medicago*, *Melilotus*, and *Lupinus* is significantly higher than in other species. Similarly, the number of genes in the SVP and AGL17 subfamilies of Type-II in Prosopis exceeds that in other species.

Conversely, gene loss has also sculpted this family. TM8 genes were lost in all Fabaceae species surveyed but present in other species, such as grape and *Amborella*. FLC orthologues were mainly detected in *Glycine*, *Dalbergia* (**Figure 4**) and BS, as a sister group of AP3/PI, also lost in 6 species (*Acacia crassicarpa*, *Bauhinia championii*, *Biancaea sappan*, *Delonix regia*, *Senna siamea*, *Vicia faba*, and *Vigna umbellata*), suggesting recurrent, lineage-specific gene loss events.

### 3.4 The dualistic engine of evolution: WGD-driven conservation versus SSD-driven innovation

**Figure 5:**
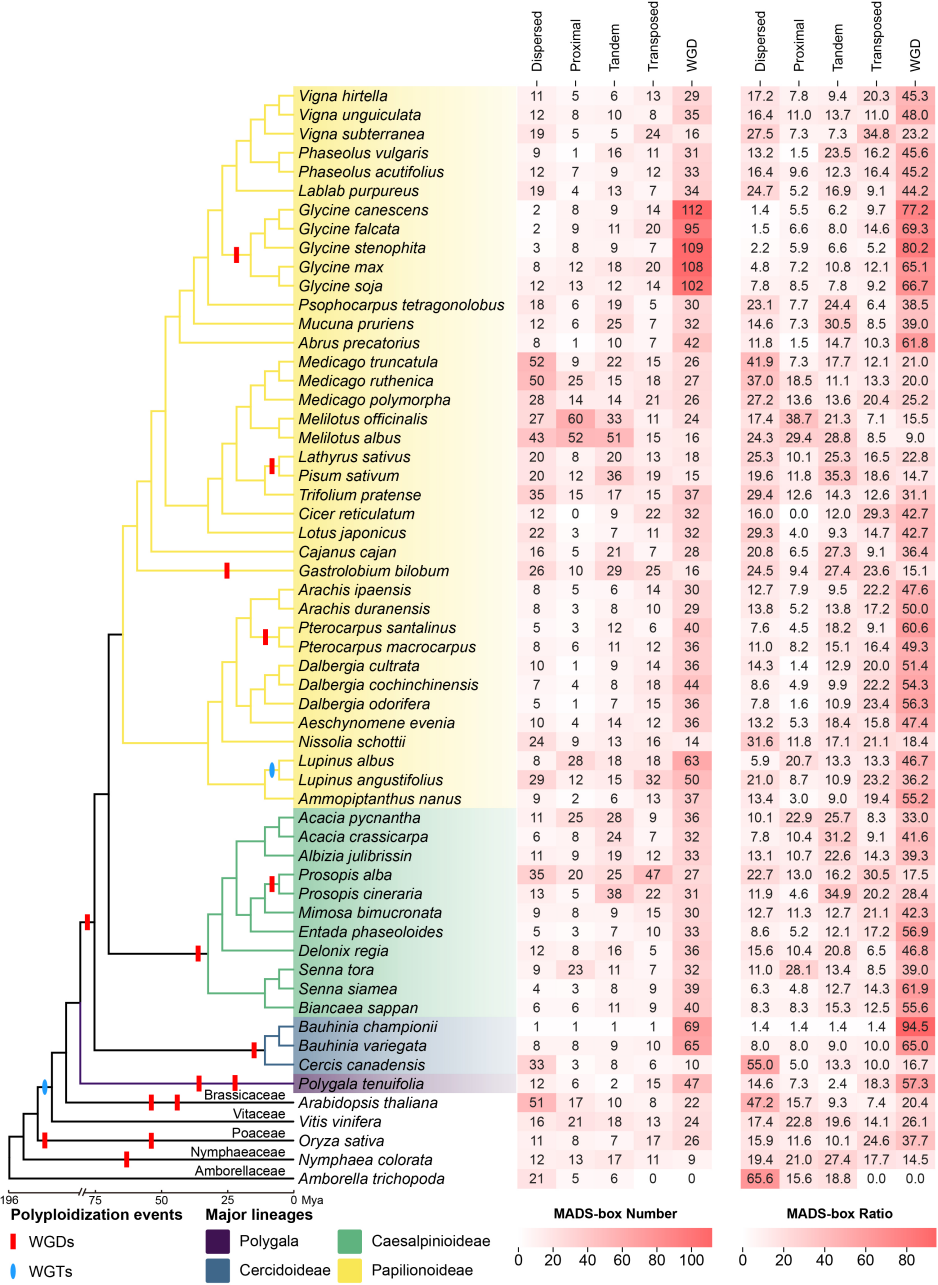
Legume species employ divergent duplication strategies to shape their MADS-box repertoires. Species-specific duplication strategies for MADS-box gene families. The left side displays the phylogenetic tree of the species, with branch lengths not directly proportional to the timescale. Bar plots show the counts (middle) and proportions (right) of genes derived from different duplication modes.

**Figure 6:**
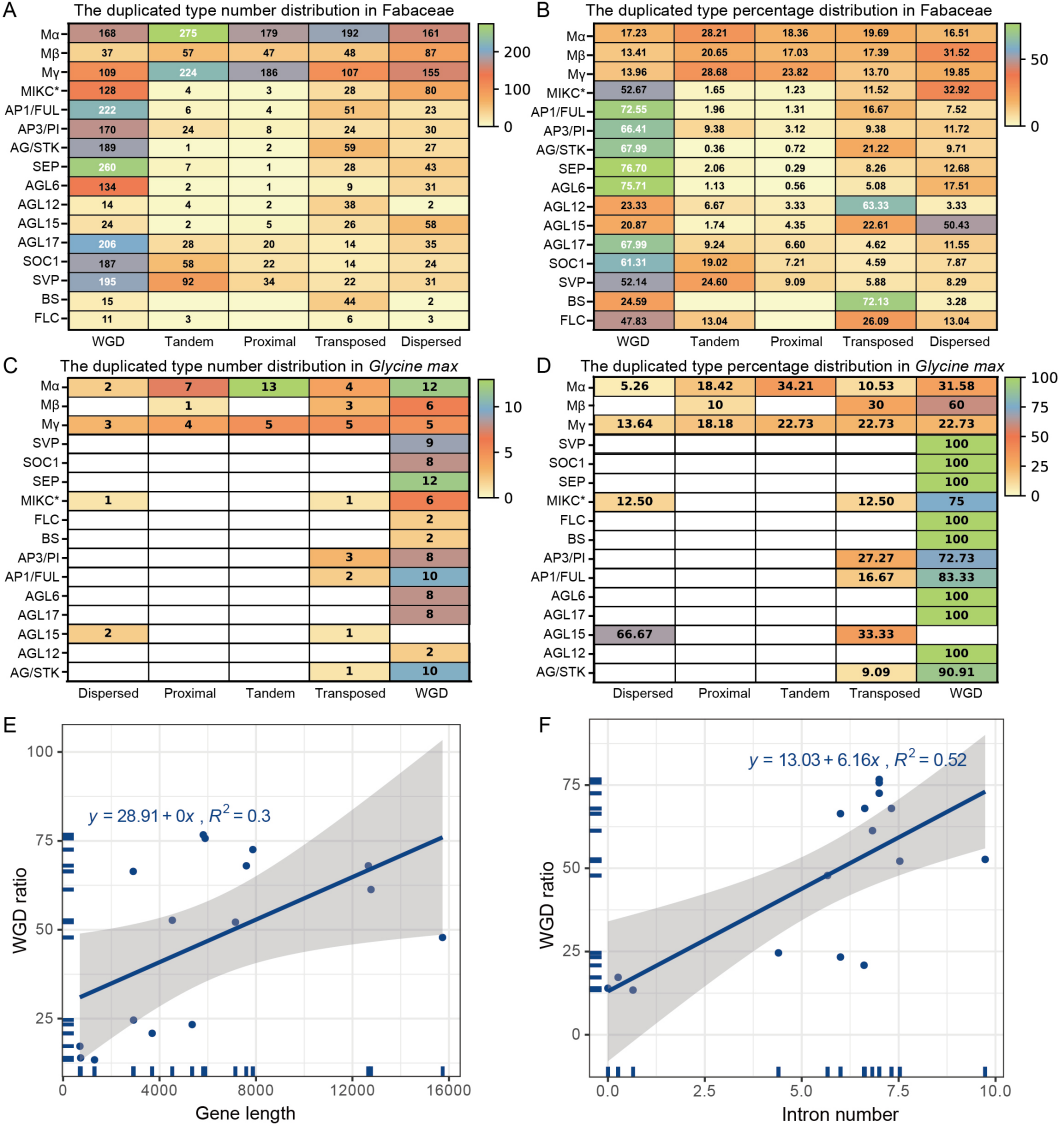
Dualistic duplication engine drives the evolution of the MADS-box family. The absolute number **(A)** and relative ratio (percentage) **(B)** of genes derived from each duplication type across 52 Fabaceae species. Example of *Glycine max* distribution in absolute number **(C)** and relative ratio **(D)**. Scatter plots showing the correlation between WGD retention ratio and gene length **(E)** or intron number **(F)** across MADS-box subfamilies.

To unravel the mechanisms behind this dynamic history, we traced the duplication origins of all genes (**Figures 5**, **6**). This revealed a striking dualistic evolutionary model. We categorized the MADS-box genes into six duplication types, including WGD, tandem, dispersed, transposed, proximal, and singleton (**Supplementary Figure S1**). Among these, WGD was the predominant type in legumes, accounting for 42.2% of the cases. This was followed by dispersed duplication (16.2%) and tandem duplication (16.1%), which exhibited comparable proportions.

#### 3.4.1 The conserved WGD-driven expansion of type II genes

The expansion of Type II subfamilies, particularly those governing fundamental developmental processes, is tightly coupled to WGD events (**Figures 5**, **6A, B**). Over 60% of the members in key subfamilies like SOC1, SEP, AP3/PI, and AG/STK originated from WGDs. The expansion of several core MADS-box families involved in the “ABCDE model” of floral organ development showed a tight coupling with WGD events. In the genus *Glycine*, which experienced a recent WGD, subfamilies such as AP1/FUL, AP3/PI, AG/STK, and SEP, as well as other subfamilies like AGL17, SOC1, and SVP, were markedly expanded compared to other legumes (**Figures 5**, **6C, D**). Within these conserved core subfamilies (e.g., AP1/FUL, AP3/PI, and SEP), genes originating from tandem duplication were extremely rare, suggesting that their expansion is under strict functional constraints and occurs primarily through WGD events (**Figures 5**, **6A, B**).

#### 3.4.2 The dynamic periphery—SSD-driven expansion of type I genes and lineage-specific type II genes

In stark contrast, all Type I subfamilies evolved dynamically, with their expansion overwhelmingly driven by a suite of SSD events—dispersed, proximal, and tandem duplications (**Figures 6A, B**). This pattern was particularly prominent in the genera *Melilotus* and *Medicago* (**Figure 5**). In *Melilotus* albus, for example, out of its large MADS-box family of 177 members, only 9.0% originated from WGD, whereas the combined contribution of dispersed, proximal, and tandem duplications was 82.5% (24.3%, 29.4%, and 28.8%, respectively. In *Medicago*, the contribution from WGD also remained low at 20%-25%, with expansion primarily driven by dispersed duplications. In *Medicago truncatula*, for example, dispersed duplications contributed 41.9%, making it the primary driver of its gene family expansion. This SSD-driven “birth-and-death” cycle also fuels innovation in specific Type II subfamilies, which are completely decoupled from WGD. A dramatic example is in the genus *Prosopis*, where the SVP subfamily expanded rapidly to 24 members via SSD duplication, accounting for 85.71% of its total 28 members (**Figure 4**; **Supplementary Figure S2**).

To explore the relationship between gene architecture and evolutionary dynamics, we analyzed the correlation between structural characteristics and WGD retention rates across all subfamilies (**Figures 6E, F**). We observed positive correlations between the WGD ratio and both gene length (**Figure 6E**) and average intron number (**Figure 6F**). These results indicate that subfamilies with higher structural complexity are more likely to be expanded via WGD, while structurally simpler subfamilies show a lower dependence on WGD.

### 3.5 Contrasting selection pressures underpin the dualistic pattern

**Figure 7:**
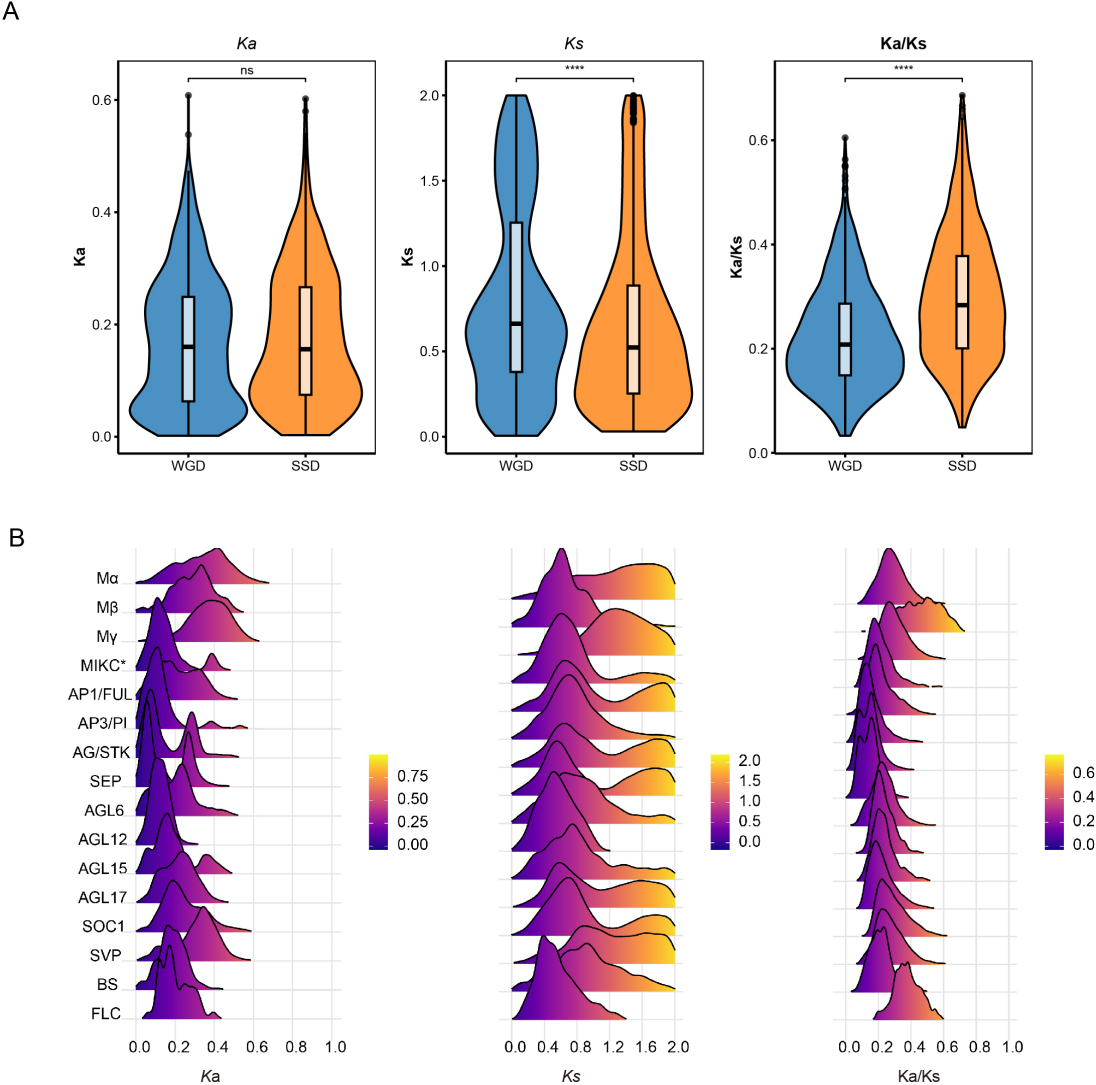
Ka/Ks analysis genes. **(A)** Statistical significance was assessed using a wilcox.test. Asterisks indicate statistical significance (*P* < 0.001). **(B)** Ridgeline plots illustrate the density distribution of *Ka*, *Ks*, and Ka/Ks ratios for duplicated gene pairs within each subfamily.

To understand the molecular basis for this dualistic pattern, we contrasted the selection pressures on 912 unique WGD-derived gene pairs and unique 635 SSD-derived gene pairs. Analysis of evolutionary rates revealed that WGD pairs exhibited significantly higher synonymous (*K*s) and non-synonymous (*K*a) substitution rates (**Figure 7A**). The median *K*s values of WGD pairs were substantially higher than those of SSD pairs (Wilcox test, *P* < 0.001), indicating that the former diverged earlier.

To reconstruct the fine-scale temporal history of these lineages, we further analyzed the distribution of *K*s across subfamilies (**Figure 7B**). This analysis revealed a clear temporal stratification. The conserved Type II subfamilies (e.g., SEP, AP1/FUL, AG/STK) exhibit a bimodal *K*s distribution. In addition to recent duplications, they display a distinct concentration of gene pairs peaks. This suggests that the expansion of the core developmental machinery was a direct legacy of this ancient polyploidy event.

Consistent with these distinct evolutionary trajectories, the analysis of selection pressure showed a completely opposite trend. The Ka/Ks ratios of WGD pairs were significantly lower than those of SSD pairs (Wilcox test, *P* < 0.001) (**Figure 7A**), suggesting the MADS-box genes from WGDs are functionally constrained by strong purifying selection, while dynamic genes from SSDs experience more relaxed constraints, affording them greater freedom to accumulate mutations and explore new functions.

### 3.6 Pangenome analysis reveals a conserved core and a dispensable periphery

**Figure 8:**
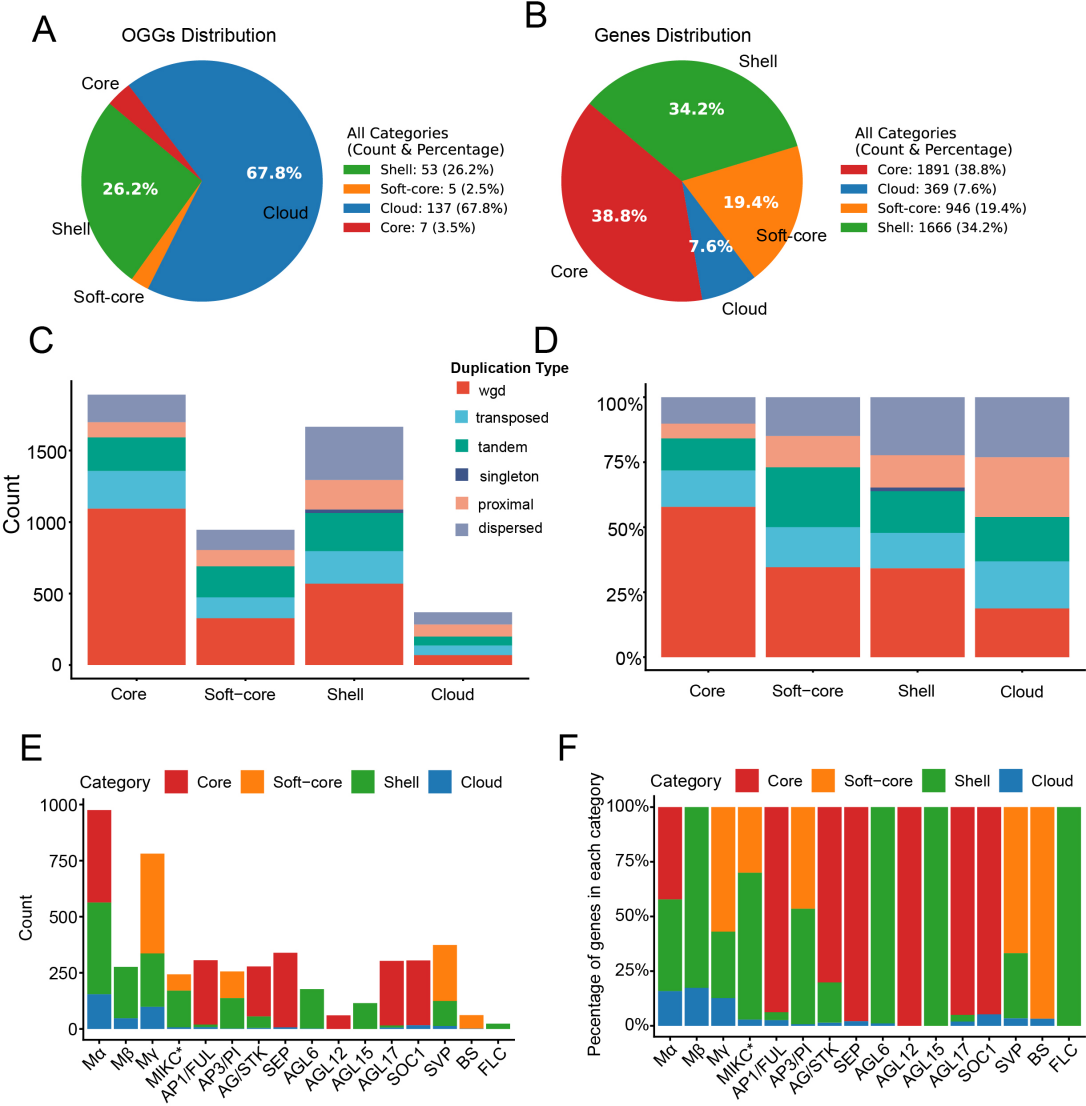
Pangenome analysis reveals a conserved core of developmental regulators and a dispensable periphery. The distribution of pan-genome family types within orthologous gene groups (OGGs) **(A)** and at the individual gene level **(B)**. The number **(C)** and percentage **(D)** Distribution of different duplication types for core, soft-core, shell, and cloud MADS-box genes. The number **(E)** and percentage **(F)** distribution of MADS-box genes in each category across different subfamilies.

The functional consequences of these divergent evolutionary paths are evident at the pangenome level (**Figure 8**). We clustered all genes into 202 orthologous gene groups (OGGs), comprising 7 core clusters (1,891 genes), 5 soft-core clusters (946 genes), 53 shell clusters (1,666 genes), 137 cloud clusters (369 genes) (**Figures 8A, B**). We further investigated the relationship between gene duplication types and the conservation of MADS-box genes in the legume pangenome (**Figures 8C, D**). We found that WGD-derived genes were enriched in the core gene set (57.80%), with their prevalence decreasing toward the cloud category (18.70%). Conversely, SSD-derived genes exhibited an opposite trend: they were most abundant in the cloud (81.30%) and least frequent in the core (42.20%). These results suggest that WGD is a major contributor to highly conserved genes, while SSD-derived genes are more often variable or accessory. In addition, our investigation of evolutionary conservation at the subfamily level revealed that the “ABCDE model” genes—*AG*/*STK*, *AGL12*, *AGL17*, *AP1*/*FUL*, *SEP*, and *SOC1*—were predominantly classified as core or soft-core genes, The near-universal presence across the 52 species highlights their critical and indispensable functional roles. For example, the high composition proportion of core genes observed in the *AGL12* (100% core genes), *SEP* (97.664%), *AGL17* (95.05%), *SOC1* (94.75%), *AP1*/*FUL* (93.79%), and *AG*/*STK* (81.39%). Conversely, subfamilies like Mβ, AGL6, AGL15 and FLC subfamilies showed lower conservation, predominantly comprised of shell genes. These results reflect their rapid turnover and variable presence across lineages, and mirror our dualistic model.

### 3.7 Expression divergence of duplicated genes in soybean

**Figure 9:**
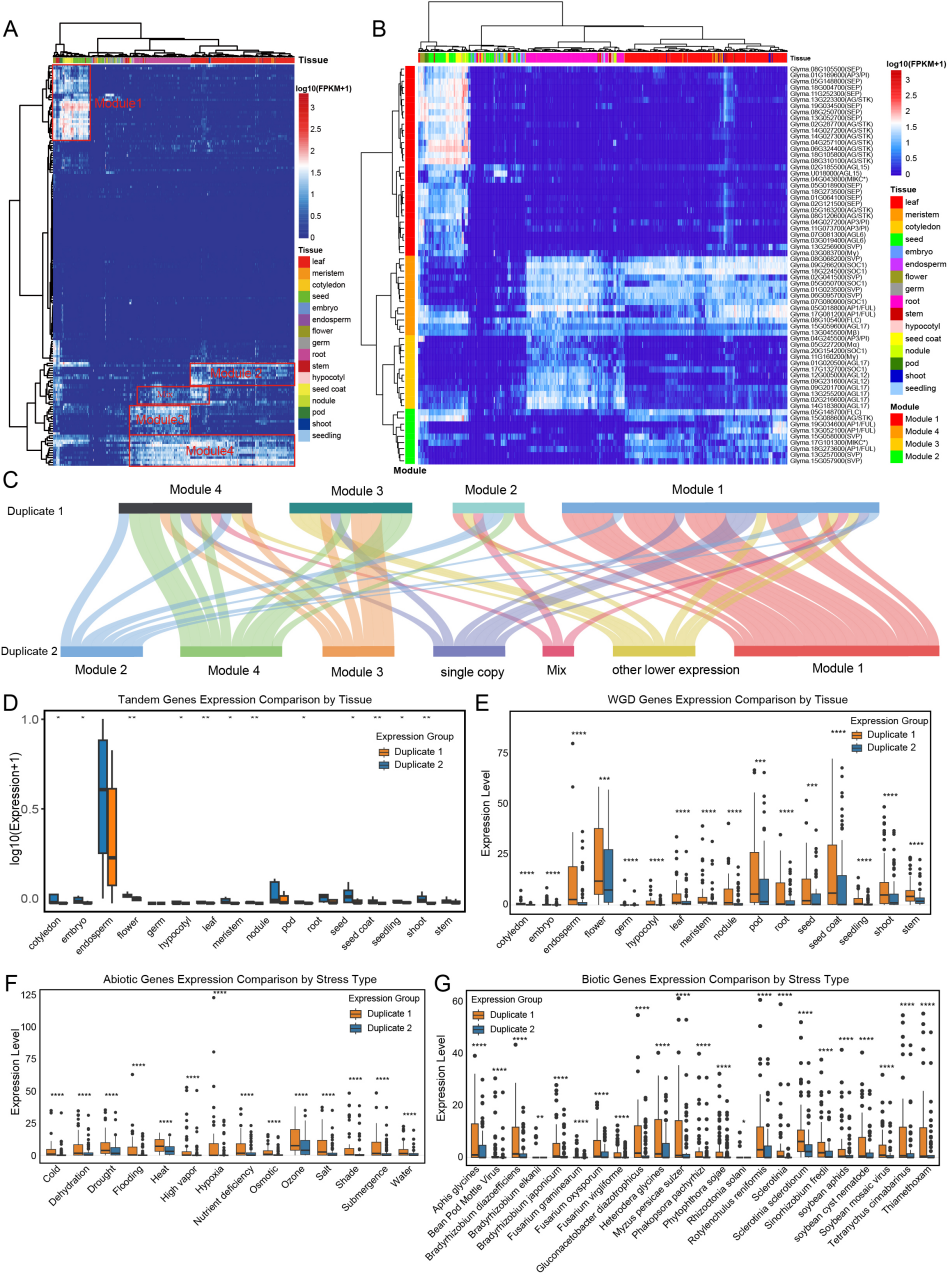
The expression atlas of soybean MADS-box genes unveils highly specialized, organ-specific regulatory modules and significant expression divergence. **(A)** Expression profiles of MADS-box genes across 3,638 samples in soybean. Clustering was performed using hierarchical clustering (Ward.D2 method, Euclidean distance) on row-scaled log2 (FPKM) values. Letters marked in the figure denote modules with higher expression levels. **(B)** Four tissue-specific expression modules identified in **(A)** are highlighted. **(C)** Distribution of duplicated gene pairs across different modules. **(D)** Expression divergence of tandem-duplicated gene pairs across various tissues. **(E)** Expression divergence of whole-genome duplication (WGD)-derived gene pairs across different tissues. **(F)** Expression divergence of WGD-derived gene pairs under abiotic stress conditions. **(G)** Expression divergence of WGD-derived gene pairs under biotic stress conditions.

A comprehensive global analysis of 3,638 soybean transcriptomes, sourced from diverse tissues under various conditions, has unveiled four distinct organ-specific modules with higher expression levels (**Figures 9A, B**). Module 1, comprised of 31 genes predominantly from the SEP and AG/STK families, displayed expression highly specific to reproductive tissues (seed coat, flower, endosperm) and but absent in leaves and roots, likely regulating seed maturation and floral organogenesis. We also found a leaf-specific module of seven genes (Module 2), likely involved in photosynthesis and flowering time control. A distinct root-specific module of 12 genes (Module 3), enriched in *AGL12* and *AGL17-like* genes, points to specialized roles in nutrient uptake and root development. Module 4 comprises 13 genes, predominantly from the SOC1 and SVP families, that are widely expressed in both roots and leaves. Our investigation into the cross-module distribution of gene duplication states revealed that the different copies of duplicated genes were systematically partitioned into different modules except for Module 1 (**Figure 9C**). This pattern implies that expression differentiation in root or leaf has occurred for these duplicated genes.

Furthermore, we focused on the expression divergence between the two main types of gene duplication: tandem duplication and whole-genome duplication (WGD). We focused on tandem duplication as the primary SSD representative because both WGD and tandem duplication are mechanistically distinct and algorithmically clear-cut duplication modes (i.e., syntenic blocks vs. local arrays). In contrast, other SSD categories like dispersed represent heterogeneous, catch-all classifications, making them less suitable for a robust comparison. Tandem duplicated pairs generally showed low expression across most tissues but were specifically upregulated in the endosperm (**Figure 9D**; **Supplementary Table S5**). In contrast, WGD-derived pairs exhibited higher and more coordinated expression across multiple tissues, particularly in flowers, seed coats, and pods, supporting their role in conserved developmental programs (**Figure 9E**). We also assessed the distribution of “high-expression, large-divergence” gene pairs—defined as those with expression levels > 10 and fold-change > 5—across different tissues. The highest number of such pairs was identified in the endosperm (17 pairs), followed by the nodule (11 pairs), and the root and seed coat (10 pairs each) (**Supplementary Table S6**).

In addition, we investigated the evolutionary and expression divergence within soybean transcriptomes in response to stress treatments. Under abiotic stress conditions, WGD-derived gene pairs sustained elevated overall expression levels, yet exhibited marked divergence among paralogs, particularly in response to ozone, salt, and drought stress (**Figure 9F**). This pattern of asymmetrical expression was even more pronounced under biotic stress. Notably, WGD-derived gene pairs demonstrated exceptionally high expression in response to infections by *Sclerotinia sclerotiorum* and *Rotylenchulus reniformis* (**Figure 9G**). Under biotic stress conditions, the average fold change of 3.24 for WGD pairs higher than the 2.76-fold change observed under abiotic stress conditions (**Supplementary Tables S7**, **S8**). This indicates that not only are asymmetric expression patterns prevalent under biotic stress, but they are also more pronounced compared to abiotic stress.

### 3.8 Explore the MADS-box genes expression in soybean under drought and salt stress treatment

**Figure 10:**
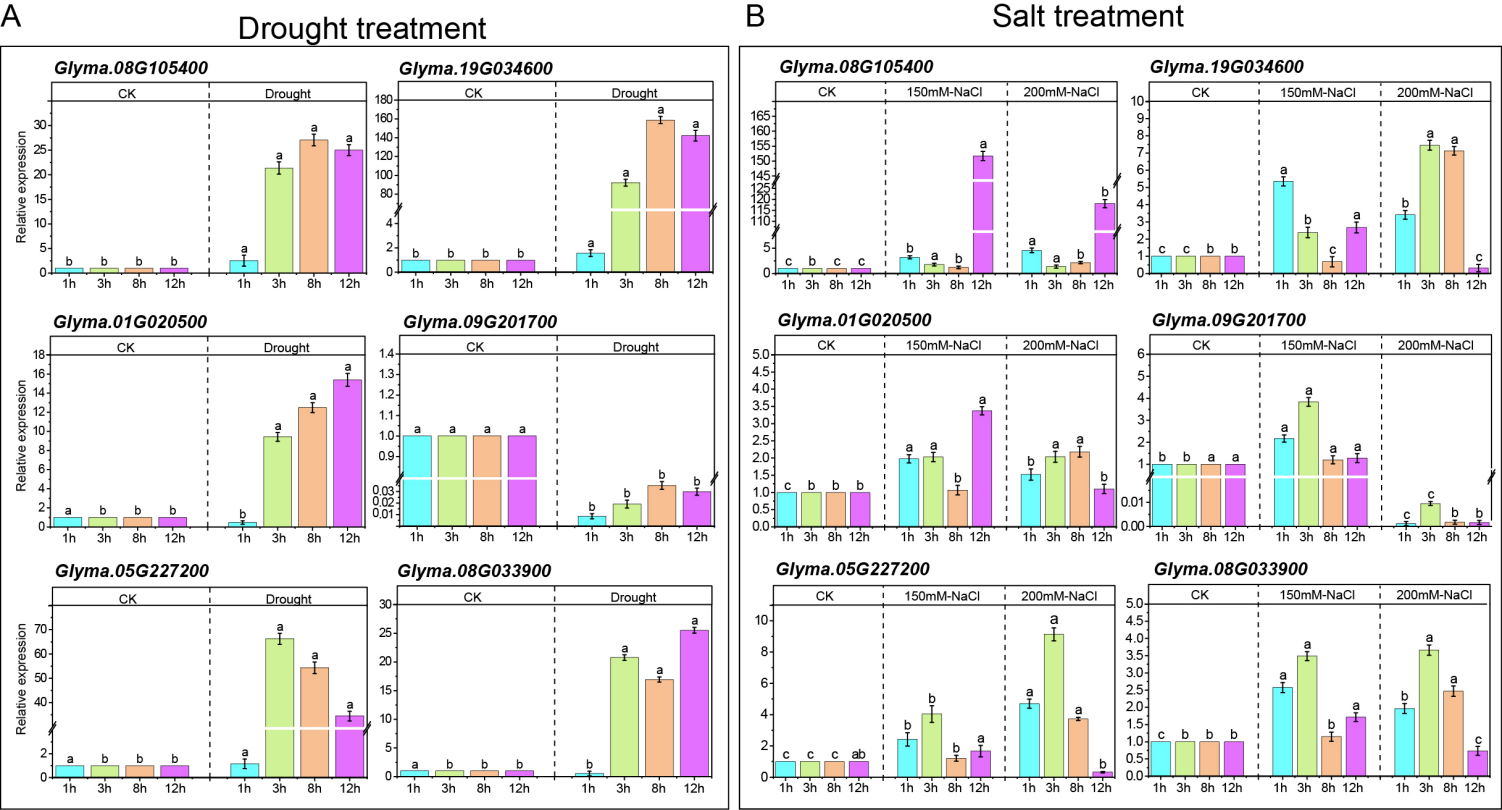
Expression of Six MADS-box genes under untreated (CK), drought, and salt conditions by qRT-PCR. **(A)** Expression of six MADS-box genes under drought stress at different time points. **(B)** Expression of six MADS-box genes under salt stress at different time points. Two different concentrations of salt (150 mM and 200 mM) were used. Data represent the mean of three independent biological replicates (± SE). Different letters above the bar charts indicate significant differences at *P* < 0.05.

We focused on six MADS-box genes responding to abiotic stress treatment (**Figure 10**). The *Glyma.08G05400*, which is broadly expressed across multiple tissues within module 4, and the *Glyma.19G034600*, which shows leaf-specific high expression in module 2. Two pairs of WGD-derived genes displaying marked expression divergence under abiotic stress—*Glyma.05G227200* vs. *Glyma.08G033900* and *Glyma.01G020500* vs. *Glyma.09G201700*—were selected for transcriptome validation. Expression analysis revealed that *Glyma.08G05400* was strongly induced under salt stress, with a more than 110-fold increase in leaves after 12 hours relative to the control. Under drought stress, this gene was significantly activated at 3 hours and peaked at 8 hours, exhibiting over 27-fold upregulation. The *Glyma.19G034600* was notably induced at 3 hours under drought stress and reached a maximum at 8 hours, with expression levels exceeding 150-fold that of the control. Among the duplicated gene pairs, both *Glyma.05G227200* and *Glyma.08G033900* were upregulated under drought stress, but their induction levels differed substantially: *Glyma.05G227200* showed over 66-fold induction, whereas *Glyma.08G033900* reached a maximum of only 25-fold. A similar divergence was observed under salt stress, where *Glyma.05G227200* was induced more than 9.13-fold compared to only 3.49-fold for *Glyma.08G033900*. We further analyzed the duplicated pair *Glyma.01G020500* and *Glyma.09G201700.* Under salt stress, both genes were comparably induced, with expression increases exceeding two-fold. Under drought stress, however, *Glyma.01G020500* was upregulated over 15-fold, while *Glyma.09G201700* was downregulated. Collectively, these results clearly demonstrate expression divergence between duplicated gene copies under stress conditions and provide experimental evidence for functional differentiation following whole-genome duplication.

## 4 Discussion

### 4.1 A Dualistic evolutionary model resolved the post-WGD paradox in legumes

Our comprehensive analysis of the MADS-box gene family across 52 Fabaceae species reveals a profound dualistic evolutionary pattern that provides a compelling solution to the post-polyploidy paradox of stability versus innovation. To anchor this model in a temporal context, our *K*s distribution analysislocalized the primary burst of WGD-derived gene retention to approximately 59 Mya. Notably, this timing places the shared legume WGD event in the immediate aftermath of the K-Pg mass extinction (Koenen et al., 2021; Vanneste et al., 2014). This temporal coincidence supports the hypothesis that polyploidy served as a critical survival strategy, providing the ancestral legume lineage with a surplus of genetic raw material to rapidly colonize ecological niches left vacant by the extinction event.

Crucially, however, the explosive radiation of legumes following this K-Pg boundary WGD was not driven by a monolithic expansion of its master developmental regulators. Instead, we propose a two-speed evolutionary architecture: a conserved core of Type II (MIKC) genes, safeguarded by strong purifying selection after being duplicated by WGD, provided developmental stability. Simultaneously, a dynamic periphery, primarily composed of Type I genes, rapidly evolved through SSDs under relaxed selection, offering a continuous source of genetic novelty. This division of evolutionary roles allowed the ancestral legume genome to reconstruct order from the genomic plasticity of WGD, ensuring the robustness of essential programs while fostering the adaptive potential that fueled its subsequent success.

### 4.2 Gene dosage balance as a guardian of developmental integrity

Our finding that core MIKC-type subfamilies expanded primarily via WGD provides strong support for the gene dosage balance hypothesis (Birchler and Veitia, 2014) (Birchler and Veitia, 2010; Conant et al., 2014; Shi et al., 2020). MADS-box proteins rarely act alone; they function by assembling into stoichiometrically precise multiprotein complexes, such as the heterotetramers of the floral quartet model, to regulate downstream gene networks (Sheng et al., 2019; Ye et al., 2021).

According to the dosage balance hypothesis, the random, small-scale duplication of a single component would disrupt this delicate stoichiometry, leading to non-functional complexes and deleterious phenotypes, and would thus be purged by strong purifying selection (Manzoor et al., 2024; Qiao et al., 2019; Wilson and Liberles, 2023). In contrast, a WGD event duplicates the entire network proportionally, preserving the balance and providing a viable pathway for the coordinated expansion of these interconnected regulatory modules (Clark and Donoghue, 2018; Soltis et al., 2015).

Our results, which show both the preferential retention of these genes post-WGD and the intense purifying selection acting upon them (low Ka/Ks ratio), empirically validate this theory. This demonstrates how a fundamental biochemical constraint—the need for stoichiometric balance—acts as a powerful evolutionary filter, preserving the integrity of core developmental machinery through deep time.

### 4.3 The dynamic periphery: a crucible for adaptive innovation

In stark contrast to the conserved core, the dynamic periphery driven by SSDs acts as a crucible for adaptive evolution (Defoort et al., 2019; Ezoe et al., 2021; Glover et al., 2015; Magadum et al., 2013). Our results show that Type I subfamilies, along with specific Type II lineages have undergone rapid, lineage-specific expansions via tandem and other SSDs. This mode of evolution, coupled with the relaxed purifying selection we observed, creates a “birth-and-death” scenario where new gene copies are constantly generated, providing raw material for neofunctionalization or subfunctionalization (Dort et al., 2024; Zhao et al., 2015). Crucially, our analysis suggests that this relaxed selection facilitates distinct functional trajectories depending on the lineage. For the enigmatic Type I subfamily, rapid evolution appears channeled primarily into reproductive specialization. Consistent with their preferential expression in seeds and gametophytes, the high sequence divergence of Type I genes may be driven by the parental conflict hypothesis or the need to establish rapid reproductive barriers, rather than abiotic stress tolerance in general (Mora-Garcia and Goodrich, 2000). In contrast, the functional shift towards environmental resilience is most prominent in SSD-expanded Type II lineages.

A compelling case for this environmental adaptation is the massive tandem expansion of the SVP subfamily in the arid-adapted genus *Prosopis*. As SVP homologs are known integrators of environmental signals that control flowering time, we hypothesize that this expanded repertoire of *SVP* genes may have allowed *Prosopis* to evolve a more sophisticated and resilient regulatory network to fine-tune its reproductive strategy in a harsh, unpredictable desert environment (Quesada-Traver et al., 2022; Shu et al., 2013). This dynamic nature is further reflected in the physical gene architecture itself. The structural analysis revealed that the Papilionoideae lineage possesses significantly more streamlined genes (i.e., fewer and shorter introns) compared to the more ancestral, intron-rich structures seen in Caesalpinioideae. This derived trait of genomic compaction, particularly prevalent in the Type I periphery (Bemer et al., 2010; De Bodt et al., 2003), would lower the energetic and temporal costs of transcription. Such streamlining acts as a powerful evolutionary facilitator for the rapid “birth-and-death” cycles and adaptive exploration that characterize these SSD-driven “accessory” genes. This provides a powerful example of the evolutionary chain linking genome structural variation, gene family expansion, and species-specific ecological adaptation.

### 4.4 WGD as an evolutionary starting point, not an endpoint

Our findings reshape the understanding of WGD’s role, casting it not as a deterministic endpoint but as a stochastic “starting point” that triggers divergent, contingent evolutionary trajectories. The stark contrast between *Bauhinia*, which largely retained its WGD duplicates, and its close relative *Cercis*, which lost them and relied on dispersed duplications for expansion, vividly illustrates this post-polyploidy divergence. This discrepancy likely reflects fundamental differences in genomic stability and evolutionary strategy. *Cercis* is often characterized as possessing a highly stable genome, closely resembling the ancestral legume karyotype (Hyun-oh et al., 2024). This suggests that *Cercis* may operate under a conservative evolutionary regime where gene dosage balance is strictly enforced, leading to the rapid fractionation of WGD duplicates to restore a diploid-like state.

Conversely, *Bauhinia* (and the Papilionoideae lineages) appears to have leveraged genomic plasticity, retaining a larger repertoire of WGD-derived regulators. This retention may have provided the necessary genetic modularity to evolve complex traits suited for diverse tropical environments. Thus, the fate of WGD genes is not uniform but is sculpted by lineage-specific constraints—balancing the immediate cost of genomic instability against the long-term benefit of adaptive potential.

Crucially, this phenomenon of contingent evolution is visible even at finer scales. Within the genus *Prosopis*, two closely related species employed entirely different SSDs strategies (tandem vs. transposed/dispersed) to shape their MADS-box repertoires. This demonstrates that the final architecture of a gene family is not predetermined by WGD alone. Rather, it is the product of a complex interplay between lineage-specific selective pressures and the particular modes of SSDs that predominate, confirming that the period of massive gene loss and rearrangement following a WGD is a critical window for evolutionary innovation (Ren et al., 2018; Wilson and Liberles, 2023).

### 4.5 Functional divergence and expression bias in soybean after whole-genome duplication

The functional data from our *Glycine max* case study provide a compelling, present-day validation of this evolutionary narrative. The expression patterns of duplicated genes directly reflect their divergent evolutionary origins. WGD-derived pairs within core subfamilies exhibit high, stable, and coordinated expression in key developmental organs, confirming their role as the conserved machinery (Carretero-Paulet and Fares, 2012; Zhao et al., 2020; Zhu et al., 2013). In contrast, the rampant asymmetric expression observed in many duplicated pairs, especially under abiotic and biotic stress, is a clear signature of functional divergence (Gu et al., 2004; Ha et al., 2007; Wei et al., 2025). The divergent or opposing stress responses of duplicated pairs (e.g., *Glyma.05G227200*/*Glyma.08G033900* and *Glyma.01G020500*/*Glyma.09G201700*) suggest their post-duplication subfunctionalization or neofunctionalization (Qian and Zhang, 2014; Zhao et al., 2020). This extensive expression divergence generates a reservoir of genes with novel expression patterns, providing the functional basis for the adaptive plasticity that has allowed legumes to thrive in diverse environments (De Smet et al., 2017; Ebadi et al., 2023; Ha et al., 2007; Kou et al., 2022).

### 4.6 Model boundaries, limitations, and future perspectives

It is important to note that the “Dualistic Model” proposed here represents a dominant evolutionary trend rather than a rigid dichotomy. We observed instances where specific lineages defy general rules under unique selective pressures. A prime example is the SVP subfamily (Type II) in the arid-adapted genus *Prosopis*. Although Type II genes are typically constrained to the conserved core driven by WGD, the SVP lineage in Prosopis has “crossed the boundary” into the dynamic periphery, undergoing SSD. This suggests that the structural constraints preventing Type II SSD expansion are not absolute. When the adaptive value of diversifying a specific regulator outweighs the cost of genomic instability, evolution can override these constraints.

While our study provides a comprehensive evolutionary framework, these exceptions highlight that our current insights represent a starting point rather than a conclusion. Our conclusions on the adaptive significance of specific gene expansions are primarily based on genomic correlation and expression data, while our deep transcriptomic insights are confined to Glycine max. Furthermore, our focus on a single gene family necessarily simplifies what was undoubtedly a genome-wide phenomenon. Therefore, the most critical next step is to move from evolutionary inference to direct functional validation. CRISPR/Cas9-based functional genomics will be essential for testing the key adaptive hypotheses proposed here, such as dissecting the role of the massively expanded SVP subfamily in the drought tolerance of Prosopis and characterizing the highly stress-responsive, asymmetrically expressed gene pairs from soybean to confirm their roles in climate resilience.

Beyond direct validation, our findings open avenues to address fundamental questions in legume biology and evolution. A major challenge is to unravel the enigma of the rapidly evolving Type I MADS-box genes. An even broader frontier lies in understanding how the MADS-box regulatory network co-evolved with other key legume innovations, such as symbiotic nitrogen fixation, by exploring how these master regulators of root development were potentially co-opted to enable the formation of a novel organ—the nodule.

## 5 Conclusion

In this study, we conducted a pangenomic analysis of MADS-box genes across 52 legume species, identifying 4,872 genes and reconstructing their phylogeny into 16 subfamilies. Our analysis revealed a pervasive dualistic evolutionary model driven by distinct duplication mechanisms. WGD-derived genes were primarily enriched in the conserved core genome, which includes essential floral regulators such as the “ABCDE model” genes. These WGD-derived genes are under strong purifying selection, thereby ensuring developmental stability. In contrast, SSD-derived genes dominate the variable regions of the genome. Evolving under relaxed selection, they facilitate lineage-specific innovation, as exemplified by the massive tandem expansion of the SVP clade in *Prosopis*. This dichotomy is also reflected in gene structure: structurally complex Type II genes tend to expand via WGD, while streamlined Type I genes proliferate through SSD. This structural–functional dichotomy was further validated in soybean, where WGD pairs show coordinated developmental expression, and exhibit stress-induced expression divergence. Our findings establish a unified evolutionary framework highlighting how duplication mechanisms and selection pressures jointly shape the legume MADS-box family.

## Data Availability

The original contributions presented in the study are included in the article/**Supplementary Material**. Further inquiries can be directed to the corresponding author.

## Supplementary material

Supplementary Table 1 Taxon list of species used for our analyses

Supplementary Table 2 Soybean RNA-seq sample listed used

Supplementary Table 3 List of primer sequences used in this study

Supplementary Table 4 MADS-box gene information identified in this study

Supplementary Table 5 Tandem duplicated gene mean expression in different tissues

Supplementary Table 6 WGD-derived duplicated gene mean expression in different tissues

Supplementary Table 7 WGD-derived duplicated gene mean expression in abiotics stress

Supplementary Table 8 WGD-derived duplicated gene mean expression in biotics stress

Supplementary Figure 1 Duplication type distributions for 4,872 MADS-box genes.

Supplementary Figure 2 The duplication distribution for SVP subfamily in reach species. **(A)** is the number distributions and **(B)** is the percentage.
