# Supplementary figures and images for: Dualistic MADS-box evolution forged legume diversity post-WGD
Source: Front Plant Sci. 2026 Jan 15;16:1740598. doi: 10.3389/fpls.2025.1740598 (PMC12853372; doi:10.3389/fpls.2025.1740598)

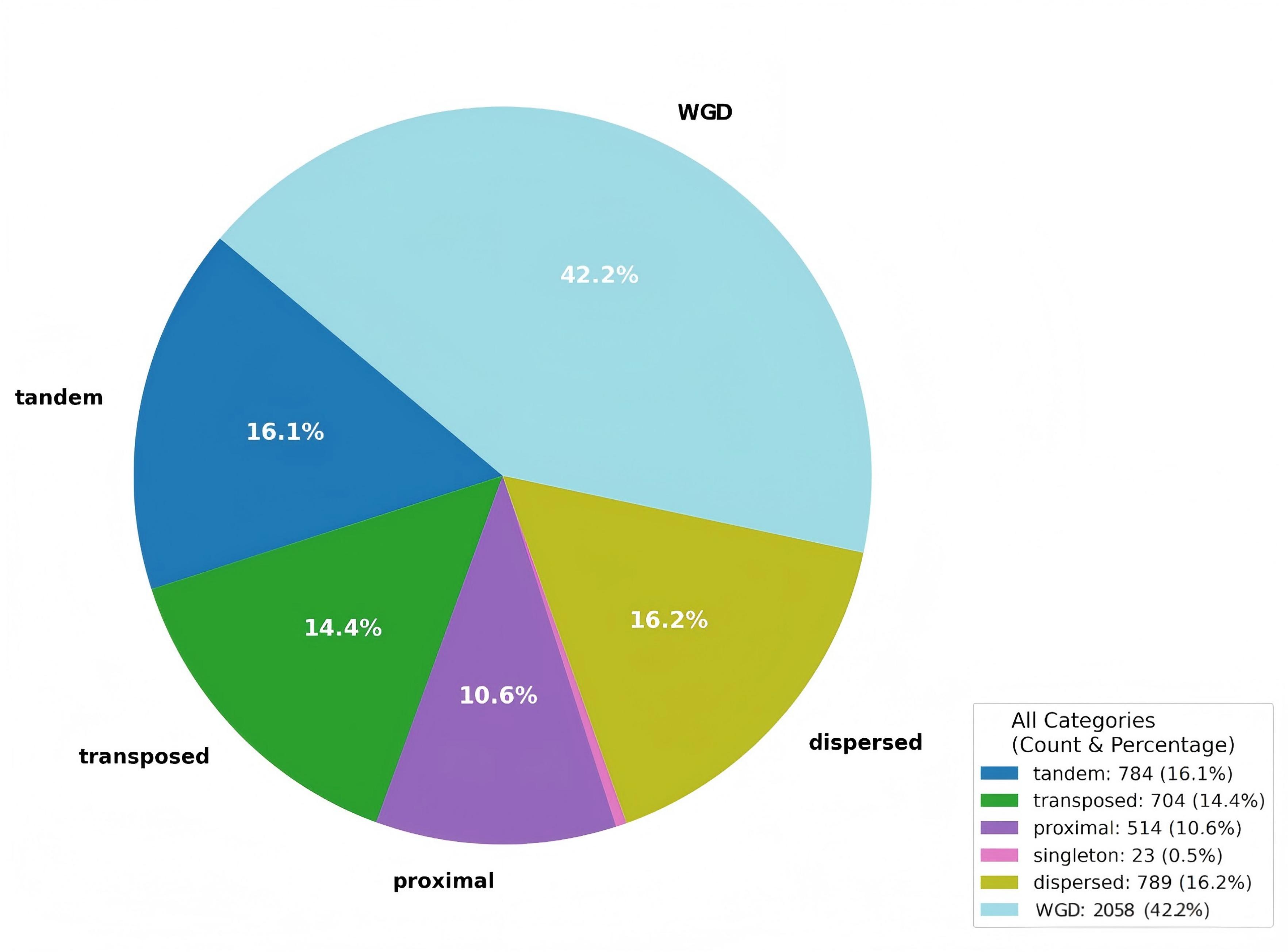

Supplement: Supplementary Figure 1 — Duplication type distributions for 4,872 MADS-box genes. [file Image1.jpg]

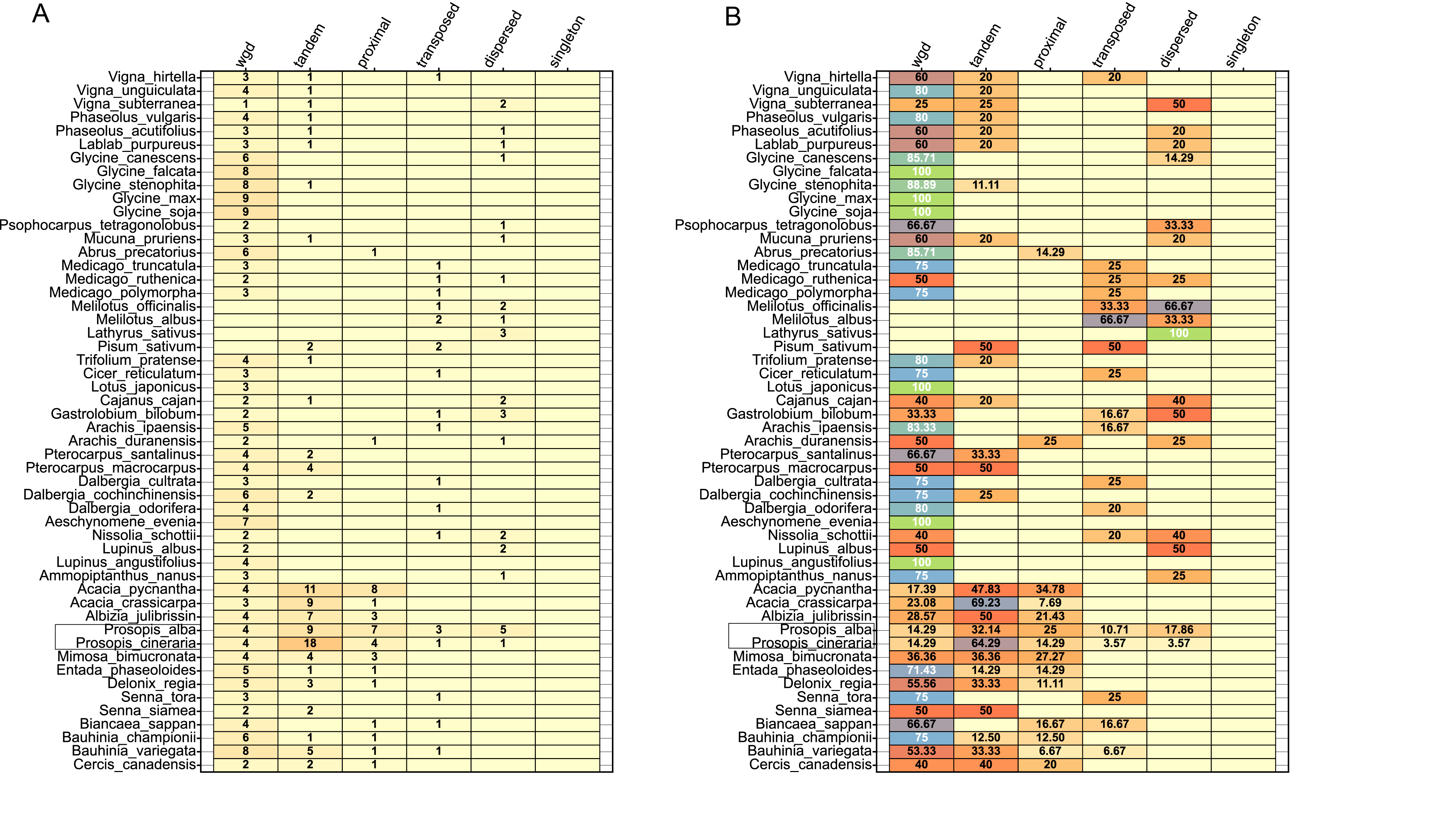

Supplement: Supplementary Figure 2 — The duplication distribution for SVP subfamily in reach species. (A) is the number distributions and (B) is the percentage. [file Image2.jpg]
